# Bidirectional Association Between Kidney Function and Atrial Fibrillation: A Population‐Based Cohort Study

**DOI:** 10.1161/JAHA.122.025303

**Published:** 2022-05-17

**Authors:** Anna C. van der Burgh, Sven Geurts, M. Arfan Ikram, Ewout J. Hoorn, Maryam Kavousi, Layal Chaker

**Affiliations:** ^1^ Department of Internal Medicine Erasmus Medical Center University Medical Center Rotterdam Rotterdam the Netherlands; ^2^ Department of Epidemiology Erasmus Medical Center University Medical Center Rotterdam Rotterdam the Netherlands

**Keywords:** arrhythmias, atrial fibrillation, bidirectional, epidemiology, kidney function, Atrial Fibrillation, Nephrology and Kidney, Epidemiology

## Abstract

**Background:**

Consensus lacks concerning a bidirectional association between kidney function and atrial fibrillation (AF), but this is crucial information for prevention/treatment efforts for both chronic kidney disease and AF. Therefore, we investigated the bidirectional association between kidney function and AF.

**Methods and Results:**

This study was a prospective cohort study including 9228 participants (mean age, 64.9 years; 57.2% women) with information on kidney function (estimated glomerular filtration rate [eGFR] based on serum creatinine [eGFRcreat], cystatin C [eGFRcys], or both [eGFRcreat‐cys], and urine albumin‐to‐creatinine ratio) and AF. Reduced kidney function was defined as eGFRcreat <60 mL/min per 1.73 m^2^. Cox proportional‐hazards, logistic regression, linear mixed, and joint models were used to investigate the association of kidney function with AF and vice versa. During follow‐up (median of 8.0 years), 780 events of incident AF occurred. Lower eGFRcys and eGFRcreat‐cys were associated with increased AF risk (hazard ratio [HR], 1.08 [95% CI, 1.03–1.14] and HR, 1.07 [95% CI, 1.01–1.14], respectively, per 10 mL/min per 1.73 m^2^ eGFR decrease). For eGFRcys and eGFRcreat‐cys, 10‐year cumulative incidence of AF was 16% (eGFR <60) and 6% (eGFR ≥60). Prevalent AF (versus no prevalent AF) was associated with 2.85 mL/min per 1.73 m^2^ lower eGFRcreat and with a faster decline of eGFRcreat with age. Prevalent AF was associated with a 1.3‐fold increased risk of incident reduced kidney function.

**Conclusions:**

Kidney function, especially eGFRcys, and AF are bidirectionally associated. There are currently no targeted prevention efforts for AF in patients with mild chronic kidney disease and vice versa. Our results could provide the first step to improve prediction/prevention of both conditions.

**Nonstandard Abbreviations and Acronyms**
ACR: albumin‐to‐creatinine ratio
eGFRcreat: eGFR based on serum creatinine
eGFRcreat‐cys: eGFR based on serum creatinine and cystatin C
eGFRcys: eGFR based on serum cystatin C

**Clinical Perspective**

**What Is New?**

- This population‐based cohort study sheds light on the currently incompletely understood bidirectional association between kidney function and atrial fibrillation (AF) in the general population.
- An increased risk of incident AF with lower kidney function is revealed, especially when kidney function is assessed by estimated glomerular filtration rate based on serum cystatin C.
- Prevalent AF is associated with lower kidney function at baseline as well as with a lower and faster decline of kidney function with the aging of the participants.

**What Are the Clinical Implications?**

- The current findings suggest that kidney function might be a potential modifiable risk factor for AF and vice versa, knowledge that could improve the prediction and prevention of both AF and chronic kidney disease.

Atrial fibrillation (AF) and chronic kidney disease (CKD) are highly prevalent diseases.^1^, ^2^ More specifically, an estimated number of 5 million new AF cases occur annually worldwide^1^ and CKD is affecting ≈11% to 13% of the global population.^2^ Furthermore, both diseases are associated with substantial morbidity and mortality from cardiovascular and cerebrovascular disease.^3^, ^4^, ^5^ Moreover, AF and CKD share several important and potential modifiable risk factors, such as hypertension and diabetes,^6^, ^7^, ^8^, ^9^ and management of these risk factors is a cornerstone in the prevention of both diseases. However, despite efforts to prevent AF and CKD mainly by managing traditional risk factors, the prevalence of both is expected to increase in the upcoming years.^10^, ^11^ This highlights the need for identifying additional risk factors to improve the prediction and prevention of AF as well as of CKD.

Interestingly, a bidirectional association between the 2 diseases may exist, revealing the potential of kidney function to be a modifiable risk factor for AF and vice versa. However, the presence of a bidirectional association between kidney function and AF in the general population is incompletely understood. Only a small number of studies have investigated a possible bidirectional association between kidney function and AF in the general population, and conflicting results were reported.^12^, ^13^ Moreover, previous population‐based studies investigating the association between kidney function and AF have several limitations, including calculating estimated glomerular filtration rate (eGFR) based on serum creatinine only (eGFRcreat). Currently, serum creatinine is widely used in clinical practice as a marker of kidney function, although it is suggested that eGFR based on cystatin C (eGFRcys) might be a stronger predictor of cardiovascular events.^14^ Therefore, it is worth investigating both markers of kidney function (i.e., eGFRcreat and eGFRcys) to investigate the potential of both in determining AF risk. Furthermore, previous studies relied on a single assessment of kidney function by which potential variation and transient declines in kidney function over time are not taken into account, which could lead to misclassification bias.

Therefore, in this study, we aimed to investigate the bidirectional association between different assessments of kidney function and AF within the general population. Moreover, we included both single and multiple assessments of kidney function to reduce the potential bias that can occur when including single assessments of kidney function only.

## Methods

The data that support the findings of this study are available from the corresponding author upon reasonable request.

### Study Design

The Rotterdam Study is a prospective, population‐based cohort study designed to investigate the occurrence and determinants of age‐related diseases in the general population. Details regarding the design and rationale of the Rotterdam Study have been described in detail previously.^15^ In summary, the Rotterdam Study is ongoing since 1990 and includes 14 926 participants aged 45 years and older, living in Ommoord, a district in Rotterdam, the Netherlands. The study consists of 3 independent cohorts: RS‐I (Rotterdam Study cohort 1), RS‐II (Rotterdam Study cohort 2), and RS‐III (Rotterdam Study cohort 3). The original cohort, RS‐I, comprised 7983 participants aged 55 years and older. In 2000, this cohort was extended with RS‐II, including 3011 participants who had become 55 years old or moved into Ommoord since the start of the study. In 2006, the cohort was further enlarged with RS‐III, including 3392 participants aged 45 years and older who had not been invited to participate previously. Follow‐up examinations are planned every 3 to 6 years and the participants are continuously monitored for relevant outcomes, including cardiovascular disease. The Rotterdam Study complies with the Declaration of Helsinki and has been approved by the Medical Ethics Committee of the Erasmus Medical Center (registration number MEC 02.1015) and by the Dutch Ministry of Health, Welfare, and Sport (Population Screening Act [WBO], license number 1071272–159521‐PG). The Rotterdam Study Personal Registration Data collection is filed with the Erasmus Medical Center Data Protection Officer under registration number EMC1712001. The Rotterdam Study has been entered into the Netherlands National Trial Register (www.trialregister.nl) and into the World Health Organization International Clinical Trials Registry Platform (www.who.int/ictrp/network/primary/en/) under shared catalogue number NTR6831. All participants provided written informed consent to participate in the study and to have their information obtained from treating physicians.

### Study Population

Participants were eligible for inclusion if they had measurements of serum creatinine and serum cystatin C available at baseline, which was defined as the third visit of RS‐I (1997–1999), the first visit of RS‐II (2000–2001), and the first visit of RS‐III (2006–2008). In addition, information on prevalent and incident AF had to be available at baseline and during follow‐up. All participants were followed up from the day of baseline laboratory measurement to the date of incident AF, date of death, loss to follow‐up, or to the end of data collection on January 1, 2014, whichever came first.

### Assessment of Kidney Function

eGFR was calculated for serum creatinine (eGFRcreat), serum cystatin C (eGFRcys), or both (eGFRcreat‐cys), according to the Chronic Kidney Disease Epidemiology Collaboration (CKD‐EPI) formula.^16^, ^17^ Serum creatinine was measured using an enzymatic assay method and expressed in micromoles per liter (µmol/L).^18^ Serum cystatin C was measured using a particle‐enhanced immunonephelometric assay and expressed in milligrams per liter (mg/L). We categorized eGFRcreat using a cut‐off of 60 mL/min per 1.73 m^2^, because eGFRcreat <60 mL/min per 1.73 m^2^ is a well‐accepted definition for reduced kidney function in population‐based research settings.^19^ The same cut‐off was used for the categorization of eGFRcys and eGFRcreat‐cys. Assessments of eGFRcreat from the Rotterdam Study were supplemented with those of the Star‐MDC database, which is a database from a center for medical diagnostics for outpatients in the city of Rotterdam, providing multiple assessments of eGFRcreat over time.^17^ In this database, serum creatinine was determined by using an enzymatic assay method as well. Incident reduced kidney function was defined as the first time eGFRcreat dropped below 60 mL/min per 1.73 m^2^. Baseline urine albumin and creatinine were determined in timed overnight urine by a turbidimetric method and measured by a Hitachi Modular P analyzer (Roche/Hitachi Diagnostics, Mannheim, Germany).^20^, ^21^ The urine albumin‐to‐creatinine ratio (ACR) was calculated by dividing urine albumin by urine creatinine (mg/g).

### Assessment of AF

Ascertainment of prevalent and incident AF within the Rotterdam Study has been reported elsewhere in detail.^22^ In short, AF ascertainment is in accordance with the European Society of Cardiology guidelines^23^ and cases were determined using 3 methods. First, ECGs that were obtained at baseline and during follow‐up examinations at the research center were stored digitally and processed by the Modular ECG Analysis System (MEANS).^24^, ^25^ As verification of the AF diagnosis, all ECGs with a MEANS diagnosis of AF, atrial flutter, or any other rhythm disorder were independently reviewed by 2 research physicians who were blinded to the MEANS diagnosis. In case of a persisting disagreement between the coding physicians, the judgement of a cardiologist was sought and taken as decisive. Second, additional information on AF was obtained from general practitioners’ records, including their own results and the results from other physicians practicing in hospitals and outpatient clinics. Finally, information was obtained from a national registry of all hospital discharge diagnoses as well. The occurrence of AF during a serious disease resulting in death, during myocardial infarction, or during cardiac operative procedures of patients who recovered during the hospital admission was not considered as a case. These participants were censored on the date of the detection of AF. We did not distinguish between AF and atrial flutter when identifying cases, because both conditions are similar with respect to risk factors and consequences.^26^, ^27^

### Assessment of Other Covariates

Body mass index was calculated as weight in kilograms divided by height in meters squared. Information on educational level, alcohol intake, tobacco smoking, and medication use was collected during home interviews. Educational level was categorized into 4 categories: primary education, lower or intermediate general and lower vocational education, higher general and intermediate vocational education, and higher vocational education or university. The highest achieved educational level was taken as a proxy for socio‐economic status. Alcohol intake was measured in grams per day and tobacco smoking was categorized into never, past, and current smokers. The World Health Organization's *Anatomical Therapeutic Chemical* code C01 was used to define cardiac medication use. Physical activity levels were assessed with a validated adapted version of the Zutphen Physical Activity Questionnaire and the (LASA) Longitudinal Aging Study Amsterdam Physical Activity Questionnaire, and expressed in total metabolic equivalent hours per week. Systolic and diastolic blood pressure were measured twice on the right arm using a random‐zero sphygmomanometer and the mean of these measurements was taken as the final measurement. Hypertension was defined as a systolic blood pressure of at least 140 mm Hg, a diastolic blood pressure of at least 90 mm Hg, or the use of antihypertensive drugs prescribed for hypertension. Serum cholesterol levels were measured by the department of Clinical Chemistry of the Erasmus Medical Center using standard laboratory techniques. Diabetes cases were defined by a previous diagnosis of the disease, a fasting serum glucose level ≥7.0 mmol/L (126 mg/dL), a non‐fasting serum glucose level ≥11.1 mmol/L (200 mg/dL; when fasting samples were absent), or the use of blood glucose‐lowering medication. History of coronary heart disease (CHD) was defined as a history of myocardial infarction or a history of a coronary revascularization procedure.^28^ Heart failure (HF) was defined in accordance with the European Society of Cardiology guidelines as a combination of the presence of typical symptoms and signs of HF (such as shortness of breath at rest or during exertion, ankle edema, or pulmonary crepitation), confirmed by objective evidence of cardiac dysfunction or a positive response to the initiated treatment.^28^, ^29^

### Statistical Analysis

To assess the potential bidirectional association between kidney function and AF, we investigated the association between kidney function and incident AF, as well as the association between prevalent AF and kidney function. To account for missing values in the covariates (missingness for all covariates <2%, except for physical activity and alcohol use, which was <20%), multiple imputation using the Multivariate Imputation by Chained Equations package in R^30^ was performed. Data were imputed using Bayesian linear regression/predictive mean matching for continuous covariates, binary logistic regression for binary categorical covariates, polytomous logistic regression for unordered categorical covariates with >2 levels, and a proportional odds model for ordered categorical covariates with >2 levels. Five imputed data sets were generated and results for each data set were pooled to obtain single estimates. Statistical significance was considered at a two‐sided *P* value <0.05. Statistical analyses were performed using R statistical software version 3.6.3 (R‐project, Institute for Statistics and Mathematics, R Core Team [2013], Vienna, Austria).

#### Kidney Function and Incident AF

Cox proportional‐hazards models were used to obtain hazard ratios (HRs) with their 95% confidence intervals (CIs) for the associations of continuous and categorized baseline eGFRcys, eGFRcreat, and eGFRcreat‐cys with incident AF. The same approach was used to study the association between urine ACR and incident AF. ACR was not normally distributed and therefore, a natural log‐transformation was used. We added 1 mg/g to the nontransformed values to account for zero values of ACR. All HRs were reported per 10 mL/min per 1.73 m^2^ decrease in eGFR and per 1 unit increase in log ACR (mg/g). The proportional‐hazards assumption was checked using the Schoenfeld test and by assessing the Schoenfeld plot. We repeated the analyses using non‐transformed ACR and HRs were reported per 1 mg/g increase in ACR. Participants with prevalent AF were excluded in the analyses regarding incident AF. Primary models were adjusted for age, sex, and Rotterdam Study cohort. We additionally adjusted for the potential confounders, educational level, body mass index, smoking, alcohol, serum total cholesterol, diabetes, physical activity, and cardiac medication use in a second model, and for the potential confounders that could also act as mediators, hypertension, history of CHD, and history of HF in a third model. Age‐ and sex‐adjusted cumulative incidences for categorized (cut‐off 60 mL/min per 1.73 m^2^) eGFRcys and eGFRcreat‐cys were extracted from standardized Cox proportional‐hazards models. Joint models were used to study the association between repeated assessments of eGFRcreat only and the risk of incident AF, because no repeated measurements of serum cystatin C were available. The longitudinal submodel was defined as a linear mixed effects model. The fixed effects in the longitudinal submodel included age, sex, and Rotterdam Study cohort, and the random effects included a random intercept and linear random slopes (i.e. of time). The survival submodel was defined as a Cox proportional‐hazards model and analyses were performed using the same models to adjust for confounders as described above. The joint model was fit under a maximum likelihood approach. Pre‐defined stratification by age and sex was performed and interaction terms of these variables with the 3 GFR estimates were used to assess effect modification. A *P* value for interaction <0.10 was considered to be statistically significant.

In sensitivity analyses, we added age, body mass index, and smoking as time‐varying covariates to the abovementioned models instead of only the baseline assessments of these covariates. In addition, we restricted the analyses to participants with eGFRcreat <120 mL/min per 1.73 m^2^ and excluded participants with prevalent CHD and HF. We also excluded participants with both prevalent and incident CHD and HF, and as a separate sensitivity analysis, we added CHD and HF as time‐varying covariates to the second model. Furthermore, we excluded the first 2 and 4 years of follow‐up to assess the possibility of reverse causality.

#### Prevalent AF and Kidney Function

Linear regression models were used to study the associations between prevalent AF and baseline levels of eGFRcys, eGFRcreat, and eGFRcreat‐cys, and linear mixed models were used to investigate the trajectories of eGFRcreat in participants with and without prevalent AF. The same 3 models to adjust for covariates as described for the analyses regarding kidney function and incident AF were used. Age was used as the time variable in the linear mixed models. First, all linear mixed models included prevalent AF as determinant and no interaction term between prevalent AF and age. Second, the analyses were repeated and an interaction term between prevalent AF and age was added to the models. The association between prevalent AF and eGFRcreat on average with age is presented by the effect estimate of prevalent AF, whereas the association between prevalent AF and change in eGFRcreat with age is represented by the effect estimate of the interaction term. Cox proportional‐hazards models were used to study the association between prevalent AF and reduced kidney function, and cases with prevalent reduced kidney function were excluded for these analyses. In a sensitivity analysis, we excluded participants with incident AF during follow‐up.

## Results

### Kidney Function and Incident AF

For the incident AF analyses, we included 9288 participants with a mean age of 64.9 years, of whom 57.2% were women (Table 1). A total of 780 cases of incident AF occurred during a median follow‐up time of 8.0 years (interquartile range, 6.1–13.3 years), with an incidence rate of 8.9 per 1000 person‐years. The total number of repeated assessments of eGFR of all participants included in the analyses regarding incident AF was 55 917, with a median of 4 assessments per participant. Lower levels of baseline eGFRcys and eGFRcreat‐cys were associated with an increased risk of incident AF, with an adjusted HR of 1.08 (95% CI, 1.03–1.14) and an adjusted HR of 1.07 (95% CI, 1.01–1.14), respectively, per 10 mL/min per 1.73 m^2^ decrease in eGFR (Table 2). Additional adjustment for hypertension, CHD, and HF did not substantially change the results (Table S1). There was no association between baseline eGFRcreat and incident AF, and similar results were reported when using repeated assessments of eGFRcreat over time (Table 2). Adding age, body mass index, and smoking as time‐varying covariates to the model, restricting the analyses to participants with eGFRcreat <120 mL/min per 1.73 m^2^, excluding participants with prevalent CHD and HF, excluding participants with prevalent and incident CHD and HF, adding CHD and HF as time‐varying covariates to the second model, and excluding the first 2 and 4 years of follow‐up did not change the risk estimates substantially (Table S2). Stratification analyses for age and sex did not show differential risks (*P* for interaction for all analyses >0.35, Table S3). Assessments of urine ACR were available in a subset of 3065 participants. No association between urine ACR and incident AF was shown (Table S4).

**Table 1:** Baseline Characteristics of the Study Population

Baseline characteristics	Participants without prevalent AF, n=9288	Participants with prevalent AF, n=409
Age, y, n=9288, n=409	64.9±9.7	73.0±10.0
Sex, women, n (%), n=9288, n=409	5317 (57.2)	185 (45.2)
Educational level, n=9205, n=406		
Primary education, n (%)	1145 (12.4)	69 (17.0)
Lower/intermediate general and lower vocational education	3718 (40.4)	174 (42.9)
Higher general and intermediate vocational education	2688 (29.2)	105 (25.9)
Higher vocational education and university	1654 (18.0)	58 (14.3)
BMI, kg/m^2^, n=9165, n=392	27.2±4.2	27.4±4.2
Systolic blood pressure, mm Hg, n=9235, n=405	140±21	142±23
Diastolic blood pressure, mm Hg, n=9235, n=405	79±11	77±13
Hypertension, n (%), n=8955, n=403	5871 (62.0)	349 (86.6)
History of diabetes, n (%), n=9288, n=409	1101 (11.9)	77 (18.8)
History of CHD, n (%), n=9240, n=405	514 (5.6)	56 (13.8)
History of HF, n (%), n=9288, n=409	184 (2.0)	87 (21.3)
Smoking, n (%), n=9190, n=402		
Current smoking	1796 (19.5)	52 (12.9)
Past smoking	4377 (47.6)	223 (55.5)
Never smoking	3017 (32.8)	127 (31.6)
Alcohol use, g/d, n=7531, n=347	5.7 (0.5–14.6)	5.1 (0.3–14.3)
eGFRcreat, mL/min per 1.73 m^2^, n=9288, n=409	81.1±14.7	70.6±18.8
eGFRcys, mL/min per 1.73 m^2^, n=9288, n=409	77.3±18.8	61.9±19.5
eGFRcreat‐cys, mL/min per 1.73 m^2^, n=9288, n=409	79.0±16.2	66.2±18.1
Serum cholesterol, mmol/L, n=9239, n=400	5.7±1.0	5.4±1.0
Cardiac medication use, n (%), n=9015, n=387	508 (5.6)	166 (42.9)

Data are presented as number (%), number (valid %), mean±SD, or median (interquartile range). Values are shown for non‐imputed data. For variables with missing data, valid % is given. AF indicates atrial fibrillation; BMI, body mass index; CHD, coronary heart disease; eGFR, estimated glomerular filtration rate; eGFRcreat, eGFR based on serum creatinine; eGFRcreat‐cys, eGFR based on serum creatinine and serum cystatin C; eGFRcys, eGFR based on serum cystatin C; and HF, heart failure.

**Table 2:** Association Between Continuous and Categorized eGFRcys, eGFRcreat, and eGFRcreat‐cys and the Risk of Incident AF (n=9288)

eGFR	AF events/Total N	HR (95% CI), Model 1	HR (95% CI), Model 2
Continuous, baseline assessment			
eGFRcys, mL/min per 1.73 m^2^	780/9288	1.11 (1.06–1.17)*	1.08 (1.03–1.14)*
eGFRcreat, mL/min per 1.73 m^2^	780/9288	1.05 (0.99–1.11)	1.04 (0.98–1.10)
eGFRcreat‐cys, mL/min per 1.73 m^2^	780/9288	1.10 (1.04–1.16)*	1.07 (1.01–1.14)*
Continuous, repeated assessments^†^			
eGFRcreat, mL/min per 1.73 m^2^	780/9288	1.03 (0.97–1.09)	1.02 (0.97–1.09)
Categorical, baseline assessment			
Categories of eGFRcys			
eGFRcys ≥60 mL/min per 1.73 m^2^	545/7599	Reference	Reference
eGFRcys <60 mL/min per 1.73 m^2^	235/1689	1.45 (1.21–1.73)*	1.37 (1.14–1.64)*
Categories of eGFRcreat			
eGFRcreat ≥60 mL/min per 1.73 m^2^	657/8422	Reference	Reference
eGFRcreat <60 mL/min per 1.73 m^2^	123 / 866	1.27 (1.03–1.56)*	1.22 (0.99–1.50)
Categories of eGFRcreat‐cys			
eGFRcreat‐cys ≥60 mL/min per 1.73 m^2^	624/8186	Reference	Reference
eGFRcreat‐cys <60 mL/min per 1.73 m^2^	156/1102	1.35 (1.11–1.64)*	1.27 (1.04–1.55)

Model 1 is adjusted for age, sex and Rotterdam Study cohort. Model 2 is additionally adjusted for educational level, BMI, smoking, alcohol, serum cholesterol, diabetes, physical activity, and use of cardiac medication. HRs are given per 10 mL/min per 1.73 m^2^ decrease in eGFR. Cox proportional‐hazards models were used to investigate the associations between continuous/categorical eGFR at baseline and incident AF. Joint models were used to investigate the association between repeated assessments of eGFRcreat and incident AF. AF indicates atrial fibrillation; BMI, body mass index; eGFR, estimated glomerular filtration rate; eGFRcreat, eGFR based on serum creatinine; eGFRcreat‐cys, eGFR based on serum creatinine and serum cystatin C; eGFRcys, eGFR based on serum cystatin C; and HR, hazard ratio.

* *P*<0.05.

^†^ Total of 55 917 repeated assessments of eGFR (median of 4 repeated assessments).

### Categories of eGFR and Incident AF

**Figure 1:**
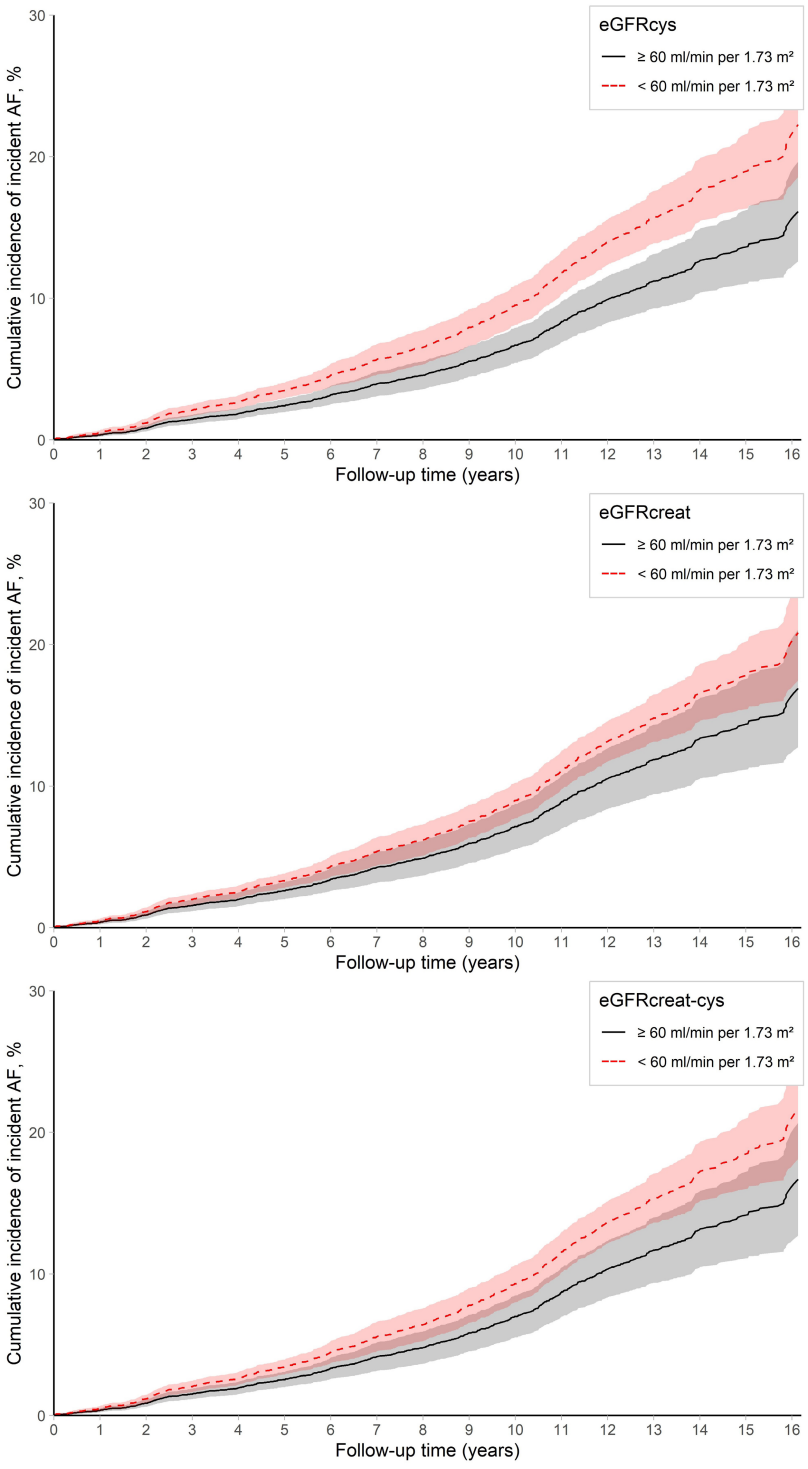
Cumulative incidence of incident AF by eGFRcys, eGFRcreat, and eGFRcreat‐cys. Cumulative incidence by eGFRcys, eGFRcreat, and eGFRcreat‐cys at baseline, adjusted for age and sex. AF indicates atrial fibrillation; eGFR, estimated glomerular filtration rate; eGFRcreat, eGFR based on serum creatinine; eGFRcreat‐cys, eGFR based on serum creatinine and serum cystatin C; and eGFRcys, eGFR based on serum cystatin C.

Categorization of eGFRcys using a cutoff of 60 mL/min per 1.73 m^2^ was associated with an increased risk of incident AF in participants with eGFRcys <60 mL/min per 1.73 m^2^ (HR, 1.37 [95% CI, 1.14–1.64]), compared with participants with eGFRcys ≥60 mL/min per 1.73 m^2^ (Table 2). Similar results were reported for eGFRcreat‐cys (HR, 1.27 [95% CI, 1.04–1.55]). In addition, cumulative incidences were higher for participants with eGFRcys <60 mL/min per 1.73 m^2^ compared with participants with eGFRcys ≥60 mL/min per 1.73 m^2^ (Figure 1). For eGFRcys, 10‐year cumulative incidence of AF was 9.5% (95% CI, 8.1–10.9) for participants with eGFR <60 mL/min per 1.73 m^2^ and 6.8% (95% CI, 5.4–7.9) for participants with eGFR ≥60 mL/min per 1.73 m^2^, with a cumulative incidence ratio of 1.40 (95% CI, 1.38–1.49). The absolute risk difference at 15 years was 5.4% (95% CI, 5.3–5.4). Categorization of eGFRcreat using a cutoff of 60 mL/min per 1.73 m^2^ was not significantly associated with an increased risk of incident AF in participants with eGFRcreat <60 mL/min per 1.73 m^2^ (HR, 1.22 [95% CI, 0.99–1.50]). When additionally adjusting for hypertension, CHD, and HF, only the association between categorized eGFRcys and incident AF remained statistically significant (data not shown).

### Prevalent AF and Kidney Function

**Figure 2:**
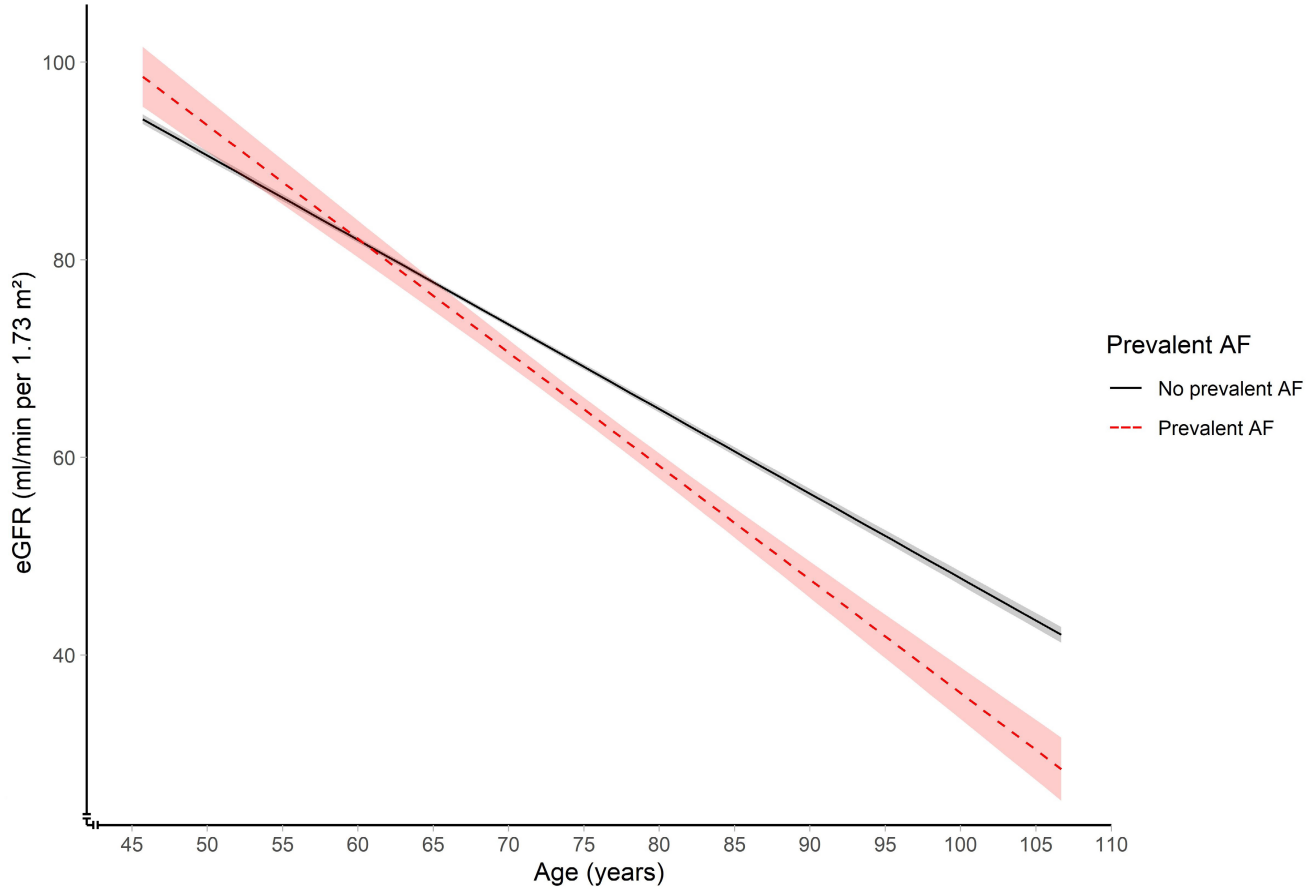
Longitudinal changes in eGFRcreat according to prevalent AF. Figure is based on an unadjusted model. *P* for interaction <0.001. Linear mixed models were used to investigate the association between prevalent AF and eGFRcreat with age. AF indicates atrial fibrillation; eGFR, estimated glomerular filtration rate; and eGFRcreat, eGFR based on serum creatinine.

For analyses regarding the association between prevalent AF and kidney function, we included 9697 participants, of whom 409 had prevalent AF at baseline (mean age, 65.3 years; 56.7% women). In a cross‐sectional analysis, eGFRcys was 4.24 mL/min per 1.73 m^2^ (95% CI, −5.68 to −2.81 mL/min per 1.73 m^2^) lower in participants with prevalent AF compared with participants without prevalent AF (Table 3). In addition, eGFRcreat and eGFRcreat‐cys were 1.93 mL/min per 1.73 m^2^ (95% CI, −3.23 to −0.63 mL/min per 1.73 m^2^) and 3.36 mL/min per 1.73 m^2^ (95% CI, −4.64 to −2.07 mL/min per 1.73 m^2^) lower in participants with prevalent AF compared with participants without prevalent AF, respectively (Table 3). The total number of repeated assessments of eGFRcreat of all participants included in the analyses with prevalent AF was 70 687, with a median of 5 assessments per participant. When studying the trajectories of eGFRcreat with age in participants with and without prevalent AF, eGFRcreat was 2.85 mL/min per 1.73 m^2^ (95% CI, −4.10 to −1.60 mL/min per 1.73 m^2^) lower in participants with prevalent AF compared with participants without prevalent AF (Table 3). Inclusion of an interaction term between AF and age also revealed a faster decline of eGFRcreat with aging in participants with prevalent AF compared with participants without prevalent AF (Figure 2). Furthermore, prevalent AF was associated with an increased risk of incident reduced kidney function, with an adjusted HR of 1.33 (95% CI, 1.12–1.58; Table 3). Excluding participants with incident AF during follow‐up did not change the risk estimates substantially (Table S5).

**Table 3:** Association Between Prevalent AF and eGFRcys, eGFRcreat, and eGFRcreat‐cys at Baseline, eGFRcreat With Age, and Incident Reduced Kidney Function (n=9697)

	Total N	β (95% CI), Model 1	β (95% CI), Model 2
Outcome: eGFR at baseline, cross‐sectional			
eGFRcys			
No prevalent AF	9288	Reference	Reference
Prevalent AF	409	−5.46 (−6.89 to −4.03)*	−4.24 (−5.68 to −2.81)*
eGFRcreat			
No prevalent AF	9288	Reference	Reference
Prevalent AF	409	−2.80 (−4.07 to −1.53)*	−1.93 (−3.23 to −0.63)*
eGFRcreat‐cys			
No prevalent AF	9288	Reference	Reference
Prevalent AF	409	−4.46 (−5.72 to −3.19)*	−3.36 (−4.64 to −2.07)*
Outcome: eGFRcreat with age, longitudinal^†^			
No prevalent AF	9288	Reference	Reference
Prevalent AF	409	−4.08 (−5.29 to −2.86)*	−2.85 (−4.10 to −1.60)*

	Events/ total n	HR (95% CI), Model 1	HR (95% CI), Model 2
Outcome: incident reduced kidney function, longitudinal^‡^			
No prevalent AF	2535/8422	Reference	Reference
Prevalent AF	157/306	1.50 (1.27 to 1.77)*	1.33 (1.12 to 1.58)*

Model 1 is adjusted for age, sex, and Rotterdam Study cohort. Model 2 is additionally adjusted for educational level, BMI, smoking, alcohol, serum cholesterol, diabetes, physical activity, and use of cardiac medication. Linear regression models were used to investigate the associations between prevalent AF and eGFR at baseline. Linear mixed models were used to investigate the association between prevalent AF and eGFRcreat with age. Cox proportional‐hazards models were used to investigate the associations between prevalent AF and incident reduced kidney function. AF indicates atrial fibrillation; BMI, body mass index; eGFR, estimated glomerular filtration rate; eGFRcreat, eGFR based on serum creatinine; eGFRcreat‐cys, eGFR based on serum creatinine and serum cystatin C; eGFRcys, eGFR based on serum cystatin C; and HR, hazard ratio.

* *P*<0.05.

^†^ Total of 70 687 repeated assessments of eGFR (median of 5 repeated assessments).

^‡^ Participants with prevalent reduced kidney function were excluded from the analysis (n=969).

## Discussion

In the current study, lower levels of eGFRcys and eGFRcreat‐cys were associated with an increased risk of incident AF. The direction of the association with eGFRcreat suggests an increased risk of incident AF with lower levels of eGFRcreat as well, although the association was less strong and not statistically significant. In cross‐sectional and longitudinal analyses, prevalent AF was associated with lower levels of all 3 GFR estimates and a faster decline of eGFRcreat with age was revealed in participants with prevalent AF.

Our findings strongly suggest the presence of a bidirectional relationship between kidney function and AF in middle‐aged and elderly individuals from the general population. Previous studies that have investigated this bidirectional association within the general population have shown conflicting results and did not include different and multiple assessments of kidney function.^12^, ^13^ One study was conducted in participants taking part in a voluntary health checkup program in Japan and reported a potential bidirectional association between kidney function assessed by eGFRcreat and AF, that is, kidney dysfunction increased the risk of incident AF and vice versa.^12^ Conversely, the second study, which included participants from a population‐based cohort of ambulatory elderly, reported no association between kidney dysfunction and both prevalent and incident AF when kidney dysfunction was defined as eGFRcreat <60 mL/min per 1.73 m^2^.^13^ They did report an association of serum cystatin C with prevalent AF, but not with incident AF. In the current study, we report that prevalent AF is associated with reduced kidney function. The availability of a high number of eGFRcreat assessments over time allowed us to investigate the trajectories of eGFRcreat with age in participants with and without prevalent AF. Our eGFRcreat trajectories revealed that eGFRcreat was lower and declined faster in participants with prevalent AF compared with participants without prevalent AF. These findings reveal that prevalent AF could be a modifiable risk factor for kidney function decline. Potentially, further studies might investigate whether early treatment of prevalent AF with appropriate drugs could prevent further deterioration of kidney function over time.

As previously mentioned, there are only a few studies investigating the bidirectional association of kidney function and AF in the general population. Several other studies investigating the association of kidney function with AF in the general population^31^, ^32^, ^33^, ^34^, ^35^, ^36^ were unidirectional and did not include multiple assessments of kidney function, which could have resulted in misclassification bias. Furthermore, some were limited in generalizability because they included only women or predominantly younger participants,^31^, ^32^, ^35^ although AF and CKD are typically diseases of older age. In addition, previous studies adjusted for different sets of confounders, which complicates an accurate comparison of their findings. In the current study, we included different and multiple assessments of kidney function and report that reduced kidney function increases the risk of incident AF in middle‐aged and elderly men and women. Moreover, the direction of the association of all 3 GFR estimates with the risk of incident AF was the same, with the strongest association reported for eGFRcys and no significant association reported for eGFRcreat. A possible explanation for our results can be found in the differences between serum cystatin C and serum creatinine, because serum cystatin C has been suggested to be a better marker of kidney function^37^ and a stronger predictor of cardiovascular events and mortality risk^14^ when compared with serum creatinine. The mechanism behind this phenomenon is not completely understood, but cystatin C appears to be less affected by age, sex, and muscle mass than serum creatinine.^14^, ^38^, ^39^, ^40^ In addition, serum cystatin C may be more sensitive for detecting small changes in eGFR.^41^

One of the mechanisms explaining the bidirectional relationship between kidney function and AF could be the presence of shared cardiovascular risk factors. However, adjusting the analyses for various cardiovascular risk factors, including in a time‐varying fashion, did not alter our risk estimates, suggesting that other and potentially more causal mechanisms could underlie the reported associations. Potential other mechanisms might be captured by the cardiorenal syndrome, an umbrella term that is used to describe the pathological interplay between the cardiovascular system and the kidneys.^42^ The pathological mechanisms involved in the cardiorenal syndrome are comprehensive and include 2 important mechanisms that could also explain the reported bidirectional association: inflammation and renin‐angiotensin aldosterone system activation. First, reduced kidney function induces a pro‐inflammatory state with a central role for inflammatory cytokines.^43^, ^44^, ^45^ Moreover, an increase in inflammatory markers and thus an inflammatory state was also shown to be related to a decrease in eGFR in the general population.^46^ Increased levels of pro‐inflammatory cytokines have also been associated with an increased risk of AF^47^, ^48^, ^49^, ^50^ and AF itself can also promote inflammation.^51^ Second, increased renin‐angiotensin aldosterone system activity is often reported in CKD,^52^ which could promote structural and electrical atrial remodeling.^52^, ^53^, ^54^ In addition, the expression of the angiotensin‐converting enzyme^55^ and the levels of plasma aldosterone^56^, ^57^, ^58^ are increased in patients with AF, suggesting an increased activity of the renin‐angiotensin aldosterone system in patients with AF as well. In turn, this increased activity could have pathological consequences for the kidneys, because especially excessive levels of aldosterone and angiotensin II could have pro‐inflammatory and pro‐fibrotic effects on the kidneys.^59^

Our study has several strengths. First, the population‐based design of the Rotterdam Study including a high number of participants with a high participation rate provides sufficient statistical power to study our research questions and makes the results generalizable to the general population of middle‐aged and elderly individuals. Second, a high number of meticulously adjudicated AF events were included in the study, due to the long follow‐up time and the extensive evaluation of AF cases. Third, the high number of eGFRcreat assessments over time allowed us to study the association between kidney function and AF in more detail and reduced the potential bias that can occur when including only a single assessment of kidney function. Although not every participant had the same number of repeated eGFRcreat assessments available, we were still able to provide valid results by using statistical methods that can handle such unbalanced data. Several limitations should be mentioned as well. First, residual confounding cannot be excluded, even though we adjusted for a wide variety of confounders. Second, eGFRcys and eGFRcreat‐cys were determined only at baseline and therefore changes in these assessments over time could not be analyzed. Third, data on urine ACR were only available in a subset of the population and only a low number of incident AF events occurred in this population. Therefore, the power to detect an association between urine ACR and incident AF could have been limited. Fourth, part of the included participants had missing values in one or more confounders, although the missingness was expected to be at random and was <2% for most confounders. Furthermore, we performed multiple imputation to account for missing values in the covariates. Finally, the Rotterdam Study includes mainly White middle‐aged and elderly subjects, which limits the generalizability of our results to other races, ethnicities, and younger populations.

In conclusion, we report an increased risk of incident AF with lower levels of eGFRcys and eGFRcreat‐cys. This reveals that kidney function, especially when assessed by eGFRcys, could be a modifiable risk factor for incident AF. In addition, we report that prevalent AF is associated with reduced kidney function, both at baseline and over time, which reveals that prevalent AF could be a modifiable risk factor for decreased kidney function. Because the prevalence of both AF and CKD is expected to increase in the upcoming years despite efforts to prevent both diseases by managing traditional risk factors, our findings may be highly clinically relevant because they could improve the prediction and prevention of both AF and CKD. In addition, it could also change the preferred treatment strategy when both conditions are present simultaneously. Although our findings suggest a bidirectional association between kidney function and AF, future studies are needed to investigate the causality of this association, for example with Mendelian randomization analyses. In addition, future studies are needed to investigate potential underlying mechanisms, first focusing on whether cardiovascular risk factors mediate the association between kidney function and AF and vice versa. Prediction studies are needed to explore whether adding eGFR, and especially eGFRcys, to screening models for incident AF could improve these models and vice versa.

## Sources of Funding

None.

## Disclosures

None.

## Supporting information

Tables S1–S5

## References

[1] Chugh SS , Havmoeller R , Narayanan K , Singh D , Rienstra M , Benjamin EJ , Gillum RF , Kim Y‐H , McAnulty JH , Zheng Z‐J , et al. Worldwide epidemiology of atrial fibrillation: a global burden of disease 2010 study. Circulation. 2014;129:837–847. doi: 10.1161/CIRCULATIONAHA.113.005119 24345399 PMC4151302

[2] Hill NR , Fatoba ST , Oke JL , Hirst JA , O'Callaghan CA , Lasserson DS , Hobbs FD . Global prevalence of chronic kidney disease – a systematic review and meta‐analysis. PLoS One. 2016;11:e0158765. doi: 10.1371/journal.pone.0158765 27383068 PMC4934905

[3] Stewart S , Hart CL , Hole DJ , McMurray JJ . A population‐based study of the long‐term risks associated with atrial fibrillation: 20‐year follow‐up of the Renfrew/Paisley study. Am J Med. 2002;113:359–364. doi: 10.1016/S0002-9343(02)01236-6 12401529

[4] Gansevoort RT , Correa‐Rotter R , Hemmelgarn BR , Jafar TH , Heerspink HJ , Mann JF , Matsushita K , Wen CP . Chronic kidney disease and cardiovascular risk: epidemiology, mechanisms, and prevention. Lancet. 2013;382:339–352. doi: 10.1016/S0140-6736(13)60595-4 23727170

[5] Toyoda K , Ninomiya T . Stroke and cerebrovascular diseases in patients with chronic kidney disease. Lancet Neurol. 2014;13:823–833. doi: 10.1016/S1474-4422(14)70026-2 25030514

[6] Benjamin EJ , Levy D , Vaziri SM , D'Agostino RB , Belanger AJ , Wolf PA . Independent risk factors for atrial fibrillation in a population‐based cohort. The Framingham Heart Study. JAMA. 1994;271:840–844. doi: 10.1001/jama.1994.03510350050036 8114238

[7] Psaty BM , Manolio TA , Kuller LH , Kronmal RA , Cushman M , Fried LP , White R , Furberg CD , Rautaharju PM . Incidence of and risk factors for atrial fibrillation in older adults. Circulation. 1997;96:2455–2461. doi: 10.1161/01.CIR.96.7.2455 9337224

[8] Yamagata K , Ishida K , Sairenchi T , Takahashi H , Ohba S , Shiigai T , Narita M , Koyama A . Risk factors for chronic kidney disease in a community‐based population: a 10‐year follow‐up study. Kidney Int. 2007;71:159–166. doi: 10.1038/sj.ki.5002017 17136030

[9] Schnabel RB , Yin X , Gona P , Larson MG , Beiser AS , McManus DD , Newton‐Cheh C , Lubitz SA , Magnani JW , Ellinor PT , et al. 50 year trends in atrial fibrillation prevalence, incidence, risk factors, and mortality in the Framingham Heart Study: a cohort study. Lancet. 2015;386:154–162. doi: 10.1016/S0140-6736(14)61774-8 25960110 PMC4553037

[10] Krijthe BP , Kunst A , Benjamin EJ , Lip GY , Franco OH , Hofman A , Witteman JC , Stricker BH , Heeringa J . Projections on the number of individuals with atrial fibrillation in the European union, from 2000 to 2060. Eur Heart J. 2013;34:2746–2751. doi: 10.1093/eurheartj/eht280 23900699 PMC3858024

[11] Hoerger TJ , Simpson SA , Yarnoff BO , Pavkov ME , Rios Burrows N , Saydah SH , Williams DE , Zhuo X . The future burden of CKD in the United States: a simulation model for the CDC CKD initiative. Am J Kidney Dis. 2015;65:403–411. doi: 10.1053/j.ajkd.2014.09.023 25468386 PMC11000251

[12] Watanabe H , Watanabe T , Sasaki S , Nagai K , Roden DM , Aizawa Y . Close bidirectional relationship between chronic kidney disease and atrial fibrillation: the Niigata preventive medicine study. Am Heart J. 2009;158:629–636. doi: 10.1016/j.ahj.2009.06.031 19781424

[13] Deo R , Katz R , Kestenbaum B , Fried L , Sarnak MJ , Psaty BM , Siscovick DS , Shlipak MG . Impaired kidney function and atrial fibrillation in elderly subjects. J Card Fail. 2010;16:55–60. doi: 10.1016/j.cardfail.2009.07.002 20123319 PMC2818049

[14] Shlipak MG , Sarnak MJ , Katz R , Fried LF , Seliger SL , Newman AB , Siscovick DS , Stehman‐Breen C . Cystatin C and the risk of death and cardiovascular events among elderly persons. N Engl J Med. 2005;352:2049–2060. doi: 10.1056/NEJMoa043161 15901858

[15] Ikram MA , Brusselle G , Ghanbari M , Goedegebure A , Ikram MK , Kavousi M , Kieboom BCT , Klaver CCW , de Knegt RJ , Luik AI , et al. Objectives, design and main findings until 2020 from the Rotterdam study. Eur J Epidemiol. 2020;35:483–517. doi: 10.1007/s10654-020-00640-5 32367290 PMC7250962

[16] Inker LA , Schmid CH , Tighiouart H , Eckfeldt JH , Feldman HI , Greene T , Kusek JW , Manzi J , Van Lente F , Zhang YL , et al. Estimating glomerular filtration rate from serum creatinine and cystatin C. N Engl J Med. 2012;367:20–29. doi: 10.1056/NEJMoa1114248 22762315 PMC4398023

[17] van der Burgh AC , Rizopoulos D , Ikram MA , Hoorn EJ , Chaker L . Determinants of the evolution of kidney function with age. Kidney Int Rep. 2021;6:3054–3063. doi: 10.1016/j.ekir.2021.10.006 34901574 PMC8640542

[18] Perrone RD , Madias NE , Levey AS . Serum creatinine as an index of renal function: new insights into old concepts. Clin Chem. 1992;38:1933–1953. doi: 10.1093/clinchem/38.10.1933 1394976

[19] Bash LD , Coresh J , Kottgen A , Parekh RS , Fulop T , Wang Y , Astor BC . Defining incident chronic kidney disease in the research setting: the ARIC study. Am J Epidemiol. 2009;170:414–424. doi: 10.1093/aje/kwp151 19535543 PMC2727177

[20] Rietveld I , Hofman A , Pols HA , van Duijn CM , Lamberts SW , Janssen JA . An insulin‐like growth factor‐I gene polymorphism modifies the risk of microalbuminuria in subjects with an abnormal glucose tolerance. Eur J Endocrinol. 2006;154:715–721. doi: 10.1530/eje.1.02144 16645019

[21] Akoudad S , Sedaghat S , Hofman A , Koudstaal PJ , van der Lugt A , Ikram MA , Vernooij MW . Kidney function and cerebral small vessel disease in the general population. Int J Stroke. 2015;10:603–608. doi: 10.1111/ijs.12465 25753173

[22] Heeringa J , van der Kuip DA , Hofman A , Kors JA , van Herpen G , Stricker BH , Stijnen T , Lip GY , Witteman JC . Prevalence, incidence and lifetime risk of atrial fibrillation: the Rotterdam study. Eur Heart J. 2006;27:949–953. doi: 10.1093/eurheartj/ehi825 16527828

[23] Hindricks G , Potpara T , Dagres N , Arbelo E , Bax JJ , Blomström‐Lundqvist C , Boriani G , Castella M , Dan G‐A , Dilaveris PE , et al. 2020 ESC guidelines for the diagnosis and management of atrial fibrillation developed in collaboration with the European Association for Cardio‐Thoracic Surgery (EACTS). Eur Heart J. 2021;42:373–498. doi: 10.1093/eurheartj/ehaa612 32860505

[24] Kors JA , van Herpen G , Wu J , Zhang Z , Prineas RJ , van Bemmel JH . Validation of a new computer program for Minnesota coding. J Electrocardiol. 1996;29:83–88. doi: 10.1016/S0022-0736(96)80025-2 9238383

[25] van Bemmel JH , Kors JA , van Herpen G . Methodology of the modular ECG analysis system means. Methods Inf Med. 1990;29:346–353. doi: 10.1055/s-0038-1634805 2233382

[26] Halligan SC , Gersh BJ , Brown RD Jr , Rosales AG , Munger TM , Shen WK , Hammill SC , Friedman PA . The natural history of lone atrial flutter. Ann Intern Med. 2004;140:265–268. doi: 10.7326/0003-4819-140-4-200402170-00008 14970149

[27] Lelorier P , Humphries KH , Krahn A , Connolly SJ , Talajic M , Green M , Sheldon R , Dorian P , Newman D , Kerr CR , et al. Prognostic differences between atrial fibrillation and atrial flutter. Am J Cardiol. 2004;93:647–649. doi: 10.1016/j.amjcard.2003.11.042 14996602

[28] Leening MJG , Kavousi M , Heeringa J , van Rooij FJA , Verkroost‐van Heemst J , Deckers JW , Mattace‐Raso FUS , Ziere G , Hofman A , Stricker BHC , et al. Methods of data collection and definitions of cardiac outcomes in the Rotterdam study. Eur J Epidemiol. 2012;27:173–185. doi: 10.1007/s10654-012-9668-8 22388767 PMC3319884

[29] Bleumink GS , Knetsch AM , Sturkenboom MC , Straus SM , Hofman A , Deckers JW , Witteman JC , Stricker BH . Quantifying the heart failure epidemic: prevalence, incidence rate, lifetime risk and prognosis of heart failure the Rotterdam study. Eur Heart J. 2004;25:1614–1619. doi: 10.1016/j.ehj.2004.06.038 15351160

[30] van Buuren S & Groothuis‐Oudshoorn K Mice: Multivariate imputation by chained equations in R 2011. 2011;45:67.

[31] Alonso A , Lopez FL , Matsushita K , Loehr LR , Agarwal SK , Chen LY , Soliman EZ , Astor BC , Coresh J . Chronic kidney disease is associated with the incidence of atrial fibrillation: the atherosclerosis risk in communities (ARIC) study. Circulation. 2011;123:2946–2953. doi: 10.1161/CIRCULATIONAHA.111.020982 21646496 PMC3139978

[32] Sandhu RK , Kurth T , Conen D , Cook NR , Ridker PM , Albert CM . Relation of renal function to risk for incident atrial fibrillation in women. Am J Cardiol. 2012;109:538–542. doi: 10.1016/j.amjcard.2011.10.006 22100025 PMC3402228

[33] Sciacqua A , Perticone M , Tripepi G , Miceli S , Tassone EJ , Grillo N , Carullo G , Sesti G , Perticone F . Renal disease and left atrial remodeling predict atrial fibrillation in patients with cardiovascular risk factors. Int J Cardiol. 2014;175:90–95. doi: 10.1016/j.ijcard.2014.04.259 24836687

[34] Xu D , Murakoshi N , Sairenchi T , Irie F , Igarashi M , Nogami A , Tomizawa T , Yamaguchi I , Yamagishi K , Iso H , et al. Anemia and reduced kidney function as risk factors for new onset of atrial fibrillation (from the Ibaraki prefectural health study). Am J Cardiol. 2015;115:328–333. 25579885 10.1016/j.amjcard.2014.10.041

[35] Bansal N , Zelnick LR , Alonso A , Benjamin EJ , de Boer IH , Deo R , Katz R , Kestenbaum B , Mathew J , Robinson‐Cohen C , et al. eGFR and albuminuria in relation to risk of incident atrial fibrillation: a meta‐analysis of the Jackson heart study, the multi‐ethnic study of atherosclerosis, and the cardiovascular health study. Clin J Am Soc Nephrol. 2017;12:1386–1398. doi: 10.2215/CJN.01860217 28798221 PMC5586568

[36] Molnar AO , Eddeen AB , Ducharme R , Garg AX , Harel Z , McCallum MK , Perl J , Wald R , Zimmerman D , Sood MM . Association of proteinuria and incident atrial fibrillation in patients with intact and reduced kidney function. J Am Heart Assoc. 2017;6. doi: 10.1161/JAHA.117.005685 PMC5586292 28684642

[37] Dharnidharka VR , Kwon C , Stevens G . Serum cystatin C is superior to serum creatinine as a marker of kidney function: a meta‐analysis. Am J Kidney Dis. 2002;40:221–226. doi: 10.1053/ajkd.2002.34487 12148093

[38] Correa S , Morrow DA , Braunwald E , Davies RY , Goodrich EL , Murphy SA , Cannon CP , O'Donoghue ML . Cystatin C for risk stratification in patients after an acute coronary syndrome. J Am Heart Assoc. 2018;7:e009077. doi: 10.1161/JAHA.118.009077 30371283 PMC6474969

[39] Stevens LA , Schmid CH , Greene T , Li L , Beck GJ , Joffe MM , Froissart M , Kusek JW , Zhang Y , Coresh J , et al. Factors other than glomerular filtration rate affect serum cystatin C levels. Kidney Int. 2009;75:652–660. doi: 10.1038/ki.2008.638 19119287 PMC4557800

[40] Stevens LA , Coresh J , Schmid CH , Feldman HI , Froissart M , Kusek J , Rossert J , Van Lente F , Bruce RD III , Zhang Y , et al. Estimating GFR using serum cystatin C alone and in combination with serum creatinine: a pooled analysis of 3,418 individuals with CKD. Am J Kidney Dis. 2008;51:395–406. doi: 10.1053/j.ajkd.2007.11.018 18295055 PMC2390827

[41] Newman DJ , Thakkar H , Edwards RG , Wilkie M , White T , Grubb AO , Price CP . Serum cystatin C measured by automated immunoassay: a more sensitive marker of changes in GFR than serum creatinine. Kidney Int. 1995;47:312–318. doi: 10.1038/ki.1995.40 7731163

[42] Rangaswami J , Bhalla V , Blair JEA , Chang TI , Costa S , Lentine KL , Lerma EV , Mezue K , Molitch M , Mullens W , et al. Cardiorenal syndrome: classification, pathophysiology, diagnosis, and treatment strategies: a scientific statement from the American Heart Association. Circulation. 2019;139:e840–e878. doi: 10.1161/CIR.0000000000000664 30852913

[43] Silverstein DM . Inflammation in chronic kidney disease: role in the progression of renal and cardiovascular disease. Pediatr Nephrol. 2009;24:1445–1452. doi: 10.1007/s00467-008-1046-0 19083024

[44] Shlipak MG , Fried LF , Crump C , Bleyer AJ , Manolio TA , Tracy RP , Furberg CD , Psaty BM . Elevations of inflammatory and procoagulant biomarkers in elderly persons with renal insufficiency. Circulation. 2003;107:87–92. doi: 10.1161/01.CIR.0000042700.48769.59 12515748

[45] Keller C , Katz R , Cushman M , Fried LF , Shlipak M . Association of kidney function with inflammatory and procoagulant markers in a diverse cohort: a cross‐sectional analysis from the multi‐ethnic study of atherosclerosis (MESA). BMC Nephrol. 2008;9:9. doi: 10.1186/1471-2369-9-9 18681974 PMC2533297

[46] Fried L , Solomon C , Shlipak M , Seliger S , Stehman‐Breen C , Bleyer AJ , Chaves P , Furberg C , Kuller L , Newman A . Inflammatory and prothrombotic markers and the progression of renal disease in elderly individuals. J Am Soc Nephrol. 2004;15:3184–3191. doi: 10.1097/01.ASN.0000146422.45434.35 15579522

[47] Amdur RL , Mukherjee M , Go A , Barrows IR , Ramezani A , Shoji J , Reilly MP , Gnanaraj J , Deo R , Roas S , et al. Interleukin‐6 is a risk factor for atrial fibrillation in chronic kidney disease: findings from the CRIC study. PLoS One. 2016;11:e0148189. doi: 10.1371/journal.pone.0148189 26840403 PMC4739587

[48] Aviles RJ , Martin DO , Apperson‐Hansen C , Houghtaling PL , Rautaharju P , Kronmal RA , Tracy RP , Van Wagoner DR , Psaty BM , Lauer MS , et al. Inflammation as a risk factor for atrial fibrillation. Circulation. 2003;108:3006–3010. doi: 10.1161/01.CIR.0000103131.70301.4F 14623805

[49] Lee SH , Chen YC , Chen YJ , Chang SL , Tai CT , Wongcharoen W , Yeh HI , Lin CI , Chen SA . Tumor necrosis factor‐alpha alters calcium handling and increases arrhythmogenesis of pulmonary vein cardiomyocytes. Life Sci. 2007;80:1806–1815. 17383682 10.1016/j.lfs.2007.02.029

[50] Ren M , Li X , Hao L , Zhong J . Role of tumor necrosis factor alpha in the pathogenesis of atrial fibrillation: a novel potential therapeutic target? Ann Med. 2015;47:316–324. doi: 10.3109/07853890.2015.1042030 25982799

[51] Hu YF , Chen YJ , Lin YJ , Chen SA . Inflammation and the pathogenesis of atrial fibrillation. Nat Rev Cardiol. 2015;12:230–243. doi: 10.1038/nrcardio.2015.2 25622848

[52] McManus DD , Saczynski JS , Ward JA , Jaggi K , Bourrell P , Darling C , Goldberg RJ . The relationship between atrial fibrillation and chronic kidney disease : epidemiologic and pathophysiologic considerations for a dual epidemic. J Atr Fibrillation. 2012;5:442. 28496745 10.4022/jafib.442 PMC5153080

[53] Nair GM , Nery PB , Redpath CJ , Birnie DH . The role of renin angiotensin system in atrial fibrillation. J Atr Fibrillation. 2014;6:972. 27957054 10.4022/jafib.972 PMC5135231

[54] Ehrlich JR , Hohnloser SH , Nattel S . Role of angiotensin system and effects of its inhibition in atrial fibrillation: clinical and experimental evidence. Eur Heart J. 2006;27:512–518. doi: 10.1093/eurheartj/ehi668 16311236

[55] Goette A , Staack T , Rocken C , Arndt M , Geller JC , Huth C , Ansorge S , Klein HU , Lendeckel U . Increased expression of extracellular signal‐regulated kinase and angiotensin‐converting enzyme in human atria during atrial fibrillation. J Am Coll Cardiol. 2000;35:1669–1677. doi: 10.1016/S0735-1097(00)00611-2 10807475

[56] Dixen U , Ravn L , Soeby‐Rasmussen C , Paulsen AW , Parner J , Frandsen E , Jensen GB . Raised plasma aldosterone and natriuretic peptides in atrial fibrillation. Cardiology. 2007;108:35–39. doi: 10.1159/000095671 16968988

[57] Goette A , Hoffmanns P , Enayati W , Meltendorf U , Geller JC , Klein HU . Effect of successful electrical cardioversion on serum aldosterone in patients with persistent atrial fibrillation. Am J Cardiol. 2001;88:906–909. doi: 10.1016/S0002-9149(01)01905-1 11676961

[58] Seccia TM , Caroccia B , Adler GK , Maiolino G , Cesari M , Rossi GP . Arterial hypertension, atrial fibrillation, and hyperaldosteronism: the triple trouble. Hypertension. 2017;69:545–550. doi: 10.1161/HYPERTENSIONAHA.116.08956 28264920 PMC5425097

[59] Brewster UC , Perazella MA . The renin‐angiotensin‐aldosterone system and the kidney: effects on kidney disease. Am J Med. 2004;116:263–272. doi: 10.1016/j.amjmed.2003.09.034 14969655

