# Supplementary material for: Bidirectional Association Between Kidney Function and Atrial Fibrillation: A Population‐Based Cohort Study
Source: J Am Heart Assoc. 2022 May 17;11(10):e025303. doi: 10.1161/JAHA.122.025303 (PMC9238570; doi:10.1161/JAHA.122.025303)
Supplement: Supplementary file 1 — Tables S1–S5 [file JAH3-11-e025303-s001.pdf]

Supplemental Material

Table S1. Association between eGFRcys, eGFRcreat, and eGFRcreat-cys at baseline and the risk of incident AF.

eGFR	AF events / Total N	HR (95% CI), Model 1	HR (95% CI), Model 2	HR (95% CI), Model 3
eGFRcys, ml/min per 1.73 m^2	780 / 9,288	1.11 (1.06-1.17)*	1.08 (1.03-1.14)*	1.07 (1.02-1.13)*
eGFRcreat, ml/min per 1.73 m^2	780 / 9,288	1.05 (0.99-1.11)	1.04 (0.98-1.10)	1.02 (0.96-1.08)
eGFRcreat-cys, ml/min per 1.73 m^2	780 / 9,288	1.10 (1.04-1.16)*	1.07 (1.01-1.14)*	1.07 (1.02-1.13)*

Model 1 is adjusted for age, sex, and Rotterdam Study cohort.
Model 2 is additionally adjusted for educational level, BMI, smoking, alcohol, serum cholesterol, DM, physical activity, and use of cardiac medication.
Model 3 is additionally adjusted for hypertension, history of CHD, and history of HF.

Hazard ratios given per 10 ml/min per 1.73 m^2 decrease in eGFR.
Cox proportional-hazards models were used to investigate the associations between eGFR at baseline and the risk of incident AF.
* $P < 0.05$.

AF = atrial fibrillation, BMI = body mass index, CHD = coronary heart disease, CI = confidence interval, DM = diabetes mellitus, eGFRcreat = estimated glomerular filtration rate (eGFR) based on serum creatinine, eGFRcreat-cys = eGFR based on serum creatinine and serum cystatin C, eGFRcys = eGFR based on serum cystatin C, HF = heart failure, HR = hazard ratio, N = number

Table S2. Sensitivity analyses for the association between eGFRcys, eGFRcreat, and eGFRcreat-cys at baseline and the risk of incident AF.

Sensitivity analysis	AF events / Total N	HR (95% CI), Model 1	HR (95% CI), Model 2
**Correcting for time-varying effects of age, BMI, and smoking**			
eGFRcys, ml/min per 1.73 m^2	780 / 9,288	1.11 (1.06-1.17)*	1.09 (1.03-1.15)*
eGFRcreat, ml/min per 1.73 m^2	780 / 9,288	1.05 (0.99-1.12)	1.04 (0.99-1.11)
eGFRcreat-cys ml/min per 1.73 m^2	780 / 9,288	1.10 (1.04-1.17)*	1.08 (1.02-1.14)*
**Restricting to participants with eGFRcreat below 120 ml/min per 1.73 m^2**			
eGFRcys, ml/min per 1.73 m^2	780 / 9,281	1.11 (1.06-1.17)*	1.08 (1.03-1.14)*
eGFRcreat, ml/min per 1.73 m^2	780 / 9,281	1.05 (0.99-1.11)	1.04 (0.98-1.10)
eGFRcreat-cys ml/min per 1.73 m^2	780 / 9,281	1.10 (1.04-1.16)*	1.07 (1.01-1.14)*
**Excluding participants with prevalent CHD and HF****			
eGFRcys, ml/min per 1.73 m^2	664 / 8,596	1.09 (1.03-1.16)*	1.07 (1.01-1.14)*
eGFRcreat, ml/min per 1.73 m^2	664 / 8,596	1.04 (0.98-1.11)	1.04 (0.97-1.10)
eGFRcreat-cys ml/min per 1.73 m^2	664 / 8,596	1.08 (1.02-1.16)*	1.07 (1.00-1.14)
**Excluding participants with prevalent and incident CHD and HF****			
eGFRcys, ml/min per 1.73 m^2	546 / 7,618	1.08 (1.01;1.15)*	1.06 (0.99;1.13)
eGFRcreat, ml/min per 1.73 m^2	546 / 7,618	1.02 (0.95;1.09)	1.01 (0.94;1.01)
eGFRcreat-cys ml/min per 1.73 m^2	546 / 7,618	1.06 (0.99;1.96)	1.04 (0.96;1.12)
**Correcting for time-varying effects of CHD and HF$**			
eGFRcys, ml/min per 1.73 m^2	780 / 9,288	*NA*	1.05 (1.00;1.10)*
eGFRcreat, ml/min per 1.73 m^2	780 / 9,288	*NA*	1.02 (0.97;1.07)
eGFRcreat-cys ml/min per 1.73 m^2	780 / 9,288	*NA*	1.04 (0.99;1.10)
**Excluding first 2 years of follow-up**			
eGFRcys, ml/min per 1.73 m^2	697 / 8,965	1.13 (1.07-1.20)*	1.10 (1.04-1.17)*
eGFRcreat, ml/min per 1.73 m^2	697 / 8,965	1.07 (1.00-1.13)*	1.05 (0.99-1.12)
eGFRcreat-cys ml/min per 1.73 m^2	697 / 8,965	1.12 (1.06-1.19)*	1.09 (1.03-1.16)*
**Excluding first 4 years of follow-up**			
eGFRcys, ml/min per 1.73 m^2	597 / 8,554	1.12 (1.05-1.19)*	1.09 (1.02-1.16)*
eGFRcreat, ml/min per 1.73 m^2	597 / 8,554	1.07 (1.01-1.15)*	1.06 (1.00-1.14)*
eGFRcreat-cys, ml/min per 1.73 m^2	597 / 8,554	1.12 (1.05-1.19)*	1.09 (1.02-1.17)*

Model 1 is adjusted for age, sex and Rotterdam Study cohort.
Model 2 is additionally adjusted for educational level, BMI, smoking, alcohol, serum cholesterol, DM, physical activity, and use of cardiac medication.
NA = not applicable.

Hazard ratios given per 10 ml/min per 1.73 m^2 decrease in eGFR.

Cox proportional-hazards models were used to investigate the associations between eGFR at baseline and the risk of incident AF.

* P < 0.05.
** The non-imputed data is used to exclude the participants with CHD and HF at baseline.
$ Model 2 is additionally adjusted for the time-varying effect of CHD and HF.

AF = atrial fibrillation, BMI = body mass index, CHD = coronary heart disease, CI = confidence interval, DM = diabetes mellitus, eGFRcreat = estimated glomerular filtration rate (eGFR) based on serum creatinine, eGFRcreat-cys = eGFR based on serum creatinine and serum cystatin C, eGFRcys = eGFR based on serum cystatin C, HF = heart failure, HR = hazard ratio, N = number

Table S3. Stratified analyses by age and sex for the association between eGFRcys, eGFRcreat, and eGFRcreat-cys at baseline and the risk of incident AF.

**Stratification variable**	**eGFR**	**AF events / Total N**	**HR (95% CI), Model 1**	**HR (95% CI), Model 2**
**Age**	eGFRcys, ml/min per 1.73 m^2			
<65		225 / 5,226	1.12 (1.02-1.23)*	1.08 (0.98-1.19)
≥65		555 / 4,062	1.11 (1.04-1.18)*	1.09 (1.02-1.16)*
	P for interaction		*0.97*	*0.93*
	eGFRcreat, ml/min per 1.73 m^2			
<65		225 / 5,226	1.06 (0.95-1.18)	1.06 (0.95-1.19)
≥65		555 / 4,062	1.04 (0.98-1.12)	1.03 (0.96-1.10)
	P for interaction		*0.92*	*0.92*
	eGFRcreat-cys, ml/min per 1.73 m^2			
<65		225 / 5,226	1.11 (1.00-1.24)	1.08 (0.97-1.21)
≥65		555 / 4,062	1.10 (1.02-1.17)*	1.07 (1.00-1.15)*
	P for interaction		*0.82*	*0.69*
**Sex**	eGFRcys, ml/min per 1.73 m^2			
Men		399 / 3,971	1.12 (1.04-1.20)*	1.10 (1.03-1.19)*
Women		381 / 5,317	1.10 (1.02-1.19)*	1.06 (0.98-1.15)
	P for interaction		*0.42*	*0.65*
	eGFRcreat, ml/min per 1.73 m^2			
Men		399 / 3,971	1.05 (0.97-1.14)	1.05 (0.97-1.14)
Women		381 / 5,317	1.05 (0.97-1.14)	1.03 (0.95-1.12)
	P for interaction		*0.46*	*0.58*
	eGFRcreat-cys, ml/min per 1.73 m^2			
Men		399 / 3,971	1.11 (1.03-1.20)*	1.10 (1.01-1.19)*
Women		381 / 5,317	1.09 (1.00-1.18)*	1.05 (0.97-1.14)
	P for interaction		*0.38*	*0.36*

Model 1 is adjusted for age, sex and Rotterdam Study cohort.
Model 2 is additionally adjusted for educational level, BMI, smoking, alcohol, serum cholesterol, DM, physical activity, and use of cardiac medication.

Hazard ratios given per 10 ml/min per 1.73m^2 decrease in eGFR.
Cox proportional-hazards models were used to investigate the associations between eGFR at baseline and the risk of incident AF.

* $P < 0.05$.

AF = atrial fibrillation, BMI = body mass index, CI = confidence interval, DM = diabetes mellitus, eGFRcreat = estimated glomerular filtration rate (eGFR) based on serum creatinine, eGFRcreat-cys = eGFR based on serum creatinine and serum cystatin C, eGFRcys = eGFR based on serum cystatin C, HR = hazard ratio, N = number

Table S4. Association between the urine ACR at baseline and the risk of incident AF (n = 3,065).

	AF events / Total N	HR (95% CI), Model 1	HR (95% CI), Model 2	HR (95% CI), Model 3
Log-transformed ACR (mg/g)	71 / 3,065	1.10 (0.88;1.38)	1.08 (0.86-1.37)	1.06 (0.83-1.34)
ACR, mg/g	71 / 3,065	1.00 (1.00;1.00)	1.00 (1.00;1.00)	1.00 (1.00;1.00)

Model 1 is adjusted for age and sex.
Model 2 is additionally adjusted for educational level, BMI, smoking, alcohol, serum cholesterol, DM, physical activity, and use of cardiac medication.
Model 3 is additionally adjusted for hypertension, history of CHD, and history of HF.

Hazard ratios given per 1 unit increase in the log ACR.
Cox proportional-hazards models were used to investigate the association between urine ACR at baseline and the risk of incident AF.

* P < 0.05.

ACR = albumin-to-creatinine ratio, AF = atrial fibrillation, BMI = body mass index, CHD = coronary heart disease, CI = confidence interval, DM = diabetes mellitus, HF = heart failure, HR = hazard ratio, N = number

Table S5. Association between prevalent AF and 1) eGFRcys, eGFRcreat, and eGFRcreat-cys at baseline, 2) eGFRcreat with age, and 3) incident reduced kidney function (n = 9,697), excluding participants with incident AF during follow-up.

	**Total N**	**Beta (95% CI), Model 1**	**Beta (95% CI), Model 2**
Outcome: eGFR at baseline (cross-sectional)			
eGFRcys			
No prevalent AF	8,508	*Reference*	*Reference*
Prevalent AF	409	-5.53 (-6.96;-4.09)*	-4.09 (-5.54;-2.65)*
eGFRcreat			
No prevalent AF	8,508	*Reference*	*Reference*
Prevalent AF	409	-2.83 (-4.11;-1.56)*	-1.79 (-3.09;-0.48)*
eGFRcreat-cys			
No prevalent AF	8,508	*Reference*	*Reference*
Prevalent AF	409	-4.46 (-5.72;-3.19)*	-3.20 (-4.49;-1.90)*
Outcome: eGFRcreat with age (longitudinal)			
	**Total N**	**Beta (95% CI), Model 1**	**Beta (95% CI), Model 2**
No prevalent AF	8,508	*Reference*	*Reference*
Prevalent AF	409	-2.51 (-3.89;-1.15)*	-1.53 (-2.95;-0.11)*
Outcome: incident reduced kidney function† (longitudinal)			
	**Events / total N**	**HR (95% CI), Model 1**	**HR (95% CI), Model 2**
No prevalent AF	2,169 / 7,765	*Reference*	*Reference*
Prevalent AF	157 / 306	1.53 (1.30-1.81)*	1.36 (1.14;1.62)*

Model 1 is adjusted for age, sex and Rotterdam Study cohort.
Model 2 is additionally adjusted for educational level, BMI, smoking, alcohol, serum cholesterol, DM, physical activity, and use of cardiac medication.

Linear regression models were used to investigate the associations between prevalent AF and eGFR at baseline. Linear mixed models were used to investigate the association prevalent AF and eGFRcreat with age. Cox proportional-hazards models were used to investigate the associations between prevalent AF and incident reduced kidney function.

* $P < 0.05$.

[†] Participants with prevalent reduced kidney function were excluded from the analysis (n = 969).

AF = atrial fibrillation, BMI = body mass index, CI = confidence interval, DM = diabetes mellitus, eGFRcreat = estimated glomerular filtration rate (eGFR) based on serum creatinine, eGFRcreat-cys = eGFR based on serum creatinine and serum cystatin C, eGFRcys = eGFR based on serum cystatin C, HR = hazard ratio, N = number
